# Endophytic *Lelliottia nimipressuralis* G24: A Potential Biocontrol Agent for Managing *Phelipanche aegyptiaca*

**DOI:** 10.3390/plants15152369

**Published:** 2026-07-31

**Authors:** Zhiyu Tang, Xin Huang, Jianjun Xu, Wenfang Luo, Siqiong Tang, Junhui Zhou, Xiang Zhang, Wei He, Kemei Li

**Affiliations:** 1 College of Agronomy, Xinjiang Agricultural University, Urumqi 830091, China; 15865802571@163.com; 2 Institute of Plant Protection, Xinjiang Uygur Autonomous Region Academy of Agricultural Sciences, Key Laboratory of Integrated Pest Management on Crops in Northwestern Oasis, Ministry of Agriculture and Rural Affairs, Xinjiang Key Laboratory of Agricultural Bio-Safety, Urumqi 830091, China; huangxin0924@126.com (X.H.); xjj72@163.com (J.X.); lf576263465@163.com (W.L.); shzutangsq@163.com (S.T.); junhuiqzhou@163.com (J.Z.); kczx268@126.com (X.Z.)

**Keywords:** endophytic bacteria, *Lelliottia nimipressuralis*, *Phelipanche aegyptiaca*, parasitic weed, biological control

## Abstract

The root parasitic weed *Phelipanche aegyptiaca* poses a serious threat to tomato production in arid regions of Northwest China, yet effective and environmentally friendly control measures remain limited. In this study, an endophytic bacterial strain G24 with strong inhibitory activity against *P. aegyptiaca* seed germination was isolated from healthy tomato tissues. In vitro germination assays showed that both the fermentation broth and cell-free supernatant of G24 completely inhibited seed germination even at 1000-fold dilution, indicating that the active metabolites are extracellularly produced or released. Pot experiments further demonstrated that G24 inoculation significantly reduced the number, fresh weight, and dry weight of parasitic broomrape nodules on tomato roots, with reductions of 69.4%, 89.0%, and 88.6%, respectively, compared with the control. Moreover, G24 significantly increased tomato stem diameter, indicating its intrinsic plant growth-promoting capacity. Although both sterile NB medium and G24 suspension increased whole-plant fresh weight, no statistically significant difference was observed between the two treatments (*p* > 0.05). The reduced leaf chlorophyll concentration per unit fresh weight in G24-treated tomatoes was a typical growth dilution effect and did not suppress overall plant performance, and no phytotoxicity was detected. Based on morphological, physiological–biochemical, and multilocus phylogenetic analyses (*16S rDNA*, *gyrB*, and *rpoB*), strain G24 was identified as *Lelliottia nimipressuralis*. As a native endophyte of tomato, *L. nimipressuralis* G24 exhibits excellent host compatibility, biocontrol efficacy, and growth-promoting activity, with no adverse effects on tomato development. This study expands the application potential of the genus *Lelliottia* in parasitic weed management and provides a novel microbial resource for the integrated and sustainable control of *P. aegyptiaca*.

## 1. Introduction

*Phelipanche aegyptiaca*, a holoparasitic root weed, produces a large, persistent seed bank and exhibits strict host specificity. It poses a severe and continuous threat to processing tomato production in Xinjiang, China, causing yield losses ranging from 30% to 80% [1]. Currently, the control of this weed mainly relies on resistant cultivar breeding, agronomic practices and chemical herbicides. Nevertheless, these conventional strategies are constrained by limited resistant germplasm, high application costs, potential phytotoxicity and the development of herbicide resistance [2]. Therefore, exploring and exploiting environmentally friendly biocontrol agents has become a promising direction for sustainable management of parasitic weeds.

Widely documented endophytic bacteria residing inside healthy plant tissues boost crop growth via nitrogen fixation, phosphate solubilization, siderophore and phytohormone secretion, induce host systemic resistance, and suppress soil-borne pathogens through antimicrobial synthesis, niche competition and activation of plant defense responses [3,4,5,6]. Multiple endophytic bacteria with dual functions of plant growth promotion and pathogen suppression have been isolated from different plants, showing great application prospects in green and sustainable crop planting systems [7]. *Bacillus amyloliquefaciens*, an endophytic species, has been widely reported to exert strong antagonistic activity against *Botrytis cinerea*, the causal agent of tomato gray mold [8]. Many culturable endophytic bacteria isolated from healthy tomato tissues include promising strains capable of controlling foliar fungal diseases such as gray mold and leaf mold [9]; indigenous tomato endophytes also strongly inhibit the tomato bacterial wilt pathogen *Ralstonia solanacearum* [10]. Moreover, numerous tomato-associated endophytic bacteria exhibit antifungal activity against phytopathogenic fungi, and their biocontrol mechanisms mainly include antibiotic production, nutrient competition, niche occupation and the induction of host systemic resistance [11]. Accordingly, isolating and screening endophytic bacteria with high antagonistic activity against *P. aegyptiaca* from healthy tomato plants is significant for establishing novel and green biocontrol technologies against this parasitic weed.

In the field of parasitic weed management, increasing attention has been paid to the exploration and application of microbial resources [2]. A variety of fungi, actinomycetes, and bacteria have been documented to suppress seed germination and haustorium formation of broomrapes (*Phelipanche* and *Orobanche* spp.) [12,13,14]. Microbial secondary metabolites have been proven to interfere with the life cycle of broomrapes and serve as key active substances for parasitic weed suppression [12,13]. Among these, *Bacillus* species have been successfully developed as biocontrol candidates to manage *P. aegyptiaca* in processing tomato production [15], with *Bacillus velezensis* recognized as an effective biocontrol agent [13]. Additionally, rhizosphere bacteria from arid environments show strong inhibitory effects on *P. aegyptiaca* seed germination, expanding the resource pool of broomrape biocontrol microbes [16].

The genus *Lelliottia*, recently reclassified from the family Enterobacteriaceae, has attracted growing attention due to its prominent plant growth-promoting traits and the ability to improve plant stress tolerance. The genus *Lelliottia* was formally proposed and established by Brady et al. (2013) based on multilocus sequence analysis [17]. Nevertheless, research regarding endophytic *Lelliottia* strains isolated from host plants for the biocontrol of *Phelipanche aegyptiaca* is still limited. Existing studies mainly focus on the growth-promoting and stress-relieving functions of *Lelliottia* strains [18,19].

Given the severe damage of *P. aegyptiaca* to Xinjiang processing tomato and the drawbacks of conventional management strategies, effective microbial biocontrol resources are needed; endophytic bacteria are core plant symbionts co-evolving within host tissues, yet most biocontrol microbes are currently isolated from bulk soil or diseased parasitic weeds, leaving host-compatible endophytes from healthy tomato largely underexplored [20,21,22,23]. Herein, we isolated endophytic bacteria from healthy tomato tissues, screened strains with strong inhibitory activity against *P. aegyptiaca* seed germination through in vitro antagonism tests, evaluated the selected strain’s suppressive effect on broomrape parasitism and its growth-promoting impact on tomato via pot trials, and identified the target strain taxonomically using molecular methods, aiming to provide novel microbial materials and theoretical support for green biocontrol of *P. aegyptiaca* utilizing high-efficiency host-specific endophytic bacterial agents.

## 2. Results

### 2.1. Screening of Bacterial Strains with High Inhibitory Activity Against Seed Germination of P. aegyptiaca

#### 2.1.1. Primary Screening for Antagonistic Bacteria

The results in Table 1 revealed significant differences in the seed germination rate and relative inhibition rate of *P. aegyptiaca* among all treatment groups (*p* < 0.05). No significant difference in germination rate was observed between the nutrient broth (NB) medium group and the blank control (CK) group. Distinct variations in inhibitory efficiency were detected among different bacterial strains. Specifically, the 100-fold diluted suspensions of strains GT-1, GT-2, GT-3, JTB-4, and G24 exhibited a strong inhibitory effect, resulting in complete inhibition of *P. aegyptiaca* seed germination. These five strains were therefore considered as a distinct group and were not included in the statistical comparison of inhibition rates. For strain TG6-2, the 100-fold diluted suspension achieved a seed germination rate of 88.09 ± 4.92% and a relative inhibition rate of 11.88%, showing a moderate inhibitory effect. In contrast, ten strains displayed weak inhibitory activity, with seed germination rates higher than or equal to 88.09%; their inhibitory effects were not significantly different from those of the CK group.

#### 2.1.2. Secondary Screening of Candidate Strains

Preliminary screening identified five strains (GT-1, GT-2, GT-3, JTB-4, and G24) with strong inhibitory effects on *P. aegyptiaca* seed germination. To evaluate their concentration-dependent activity, fermentation broths were tested at 200-, 500-, and 1000-fold dilutions, with distilled water (CK) and NB medium as controls (Table 2). No significant difference was found between CK and NB, confirming that the medium itself had no inhibitory effect and that the observed inhibition was exclusively due to the fermentation broths.

The five strains differed significantly in their inhibitory performance. Strain G24 showed the strongest inhibitory activity: the seed germination rate remained 0 and the relative inhibition rate reached 100% at all three dilution multiples, achieving complete inhibition even at the highest dilution. The inhibitory effects of GT-1, GT-2, GT-3 and JTB-4 displayed obvious concentration dependence, with activity declining as the dilution multiple increased. Among them, JTB-4 presented relatively superior inhibition, with a relative inhibition rate of 15.21% at 200-fold dilution. GT-2 ranked second in inhibitory capacity. GT-1 and GT-3 showed weak bioactivity; their relative inhibition rates dropped to only 2.93% and 1.02% at 1000-fold dilution, which were not significantly different from the control groups.

### 2.2. Inhibitory Effect of Strain G24 Fermentation Broth on Seed Germination of P. aegyptiaca in Tomato Rhizosphere

Strain G24 was selected from a collection of endophytic isolates based on its inhibitory activity against *P. aegyptiaca* seed germination. The results revealed that different treatments exerted significant effects on the germination rate of *P. aegyptiaca* seeds in the tomato rhizosphere (*p* < 0.05). The highest seed germination rate of 89.51% ± 1.68% was observed in the blank control (distilled water, CK). The germination rate in the NB medium group was 67.72% ± 4.84%, which was significantly lower than that of the CK group.

**Figure 1:**
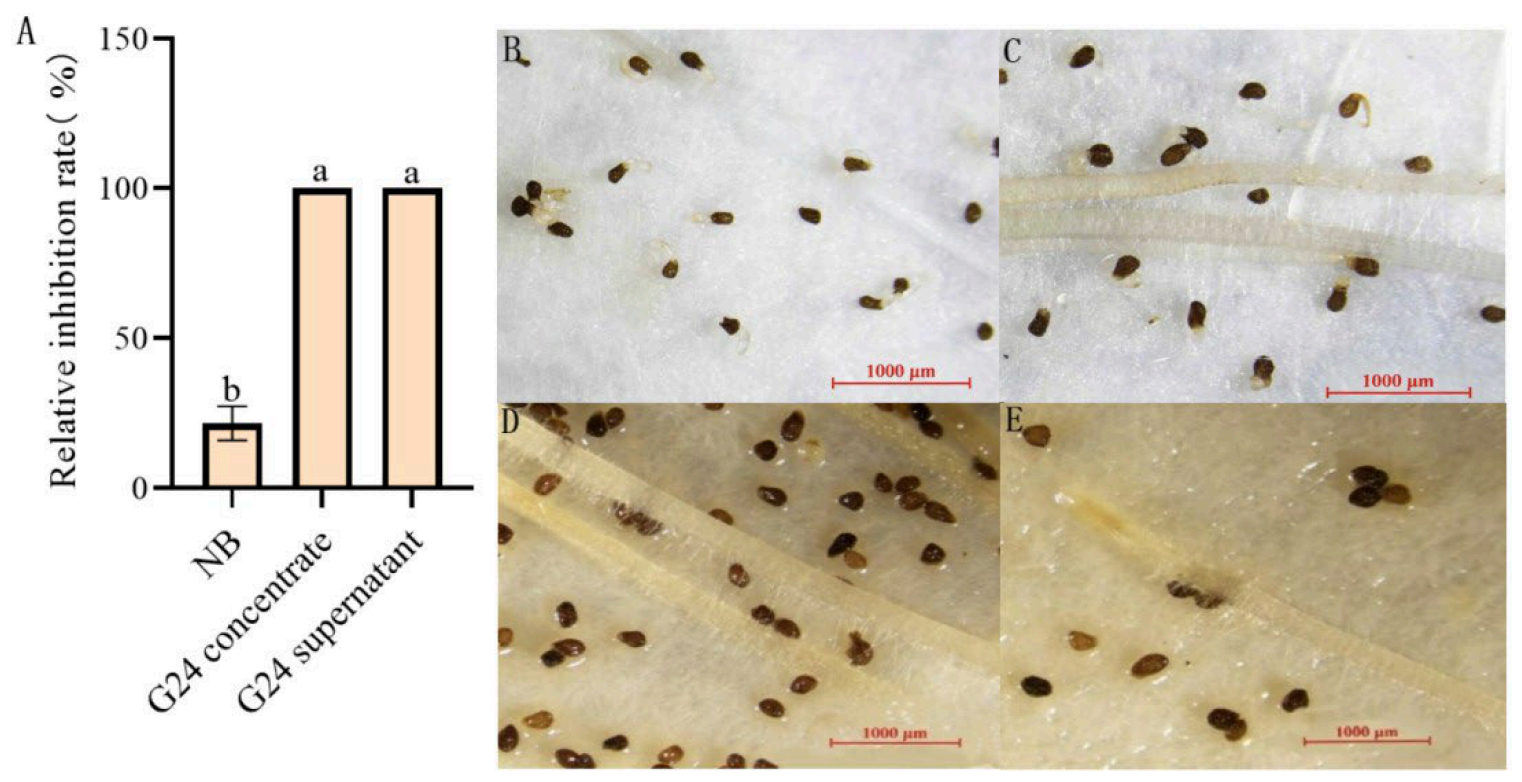
Relative inhibition rate of *P. aegyptiaca* seed germination under treatments of NB medium, G24 concentrate, and G24 supernatant (**A**); microscopic observation of broomrape seed germination in each group (**B**–**E**). (**B**), sterile water control (CK); (**C**), NB medium treatment; (**D**), G24 supernatant treatment; (**E**), G24 concentrate treatment. All seeds were cultured in the tomato rhizosphere. Values are presented as mean ± SD. Different lowercase letters above bars indicate significant differences at *p* < 0.05 according to Duncan’s test.

At a 4-fold dilution, both the original fermentation broth and supernatant of strain G24 completely inhibited seed germination, with a germination rate of 0% and an inhibition rate of 100%. Their inhibitory effects were significantly stronger than those of the CK and NB groups (*p* < 0.05) (Figure 1A).

Microscopic observations were consistent with the quantitative results. Many germinated *P. aegyptiaca* seeds with plump morphology and elongated radicles were found in the CK group (Figure 1B). Fewer germinated seeds were detected in the NB group, while most seeds still germinated normally (Figure 1C). By contrast, no germinated seeds were observed in groups treated with the original fermentation broth and supernatant of strain G24, and all seeds remained ungerminated (Figure 1D,E).

Collectively, the fermentation broth and its supernatant of strain G24 exhibited strong inhibitory activity against *P. aegyptiaca* seed germination in the tomato rhizosphere even after 4-fold dilution. The supernatant exhibited inhibitory efficacy equivalent to that of the original broth, indicating that the bioactive metabolites of strain G24 are present extracellularly, via either extracellular production or release.

### 2.3. Biocontrol Efficacy of Strain G24 Against P. aegyptiaca and Its Effects on Tomato Plant Growth

#### 2.3.1. Effects of Strain G24 on *P. aegyptiaca* Parasitism in Tomato

**Figure 2:**
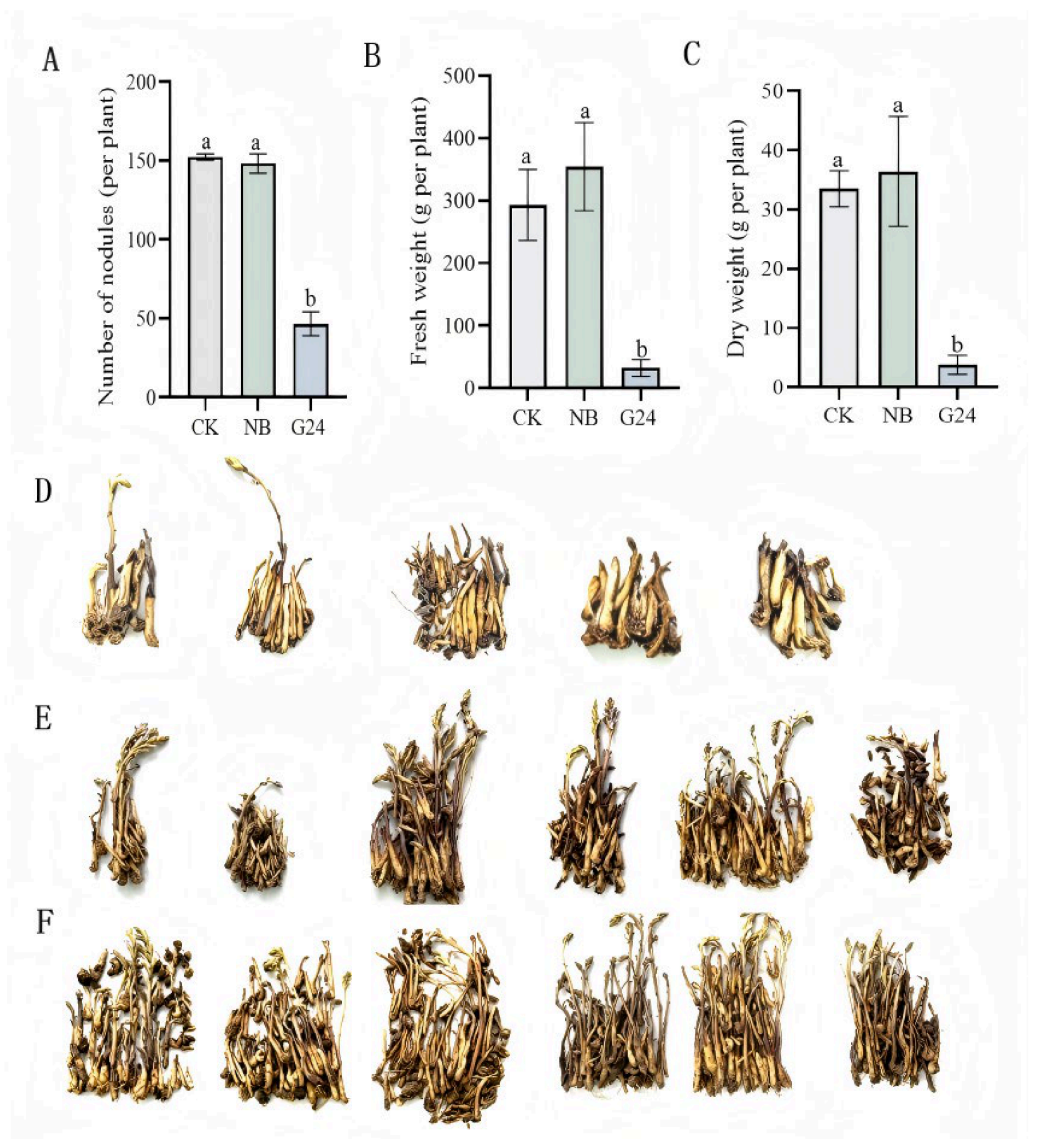
Effects of different treatments on the parasitism characteristics of *P. aegyptiaca* on tomato roots. (**A**) Number of broomrape nodules per tomato plant under CK, NB, and G24 treatments; (**B**) Fresh weight of broomrape nodules per plant under different treatments; (**C**) Dry weight of broomrape nodules per plant under different treatments; (**D**–**F**) Phenotypic observations of broomrape parasitism in the G24, NB treatment groups and CK, respectively. Values are presented as mean ± SD. Different lowercase letters above bars indicate significant differences at *p* < 0.05 according to Duncan’s test.

A pot experiment was conducted to evaluate the biocontrol efficacy of strain G24 against *P. aegyptiaca* parasitism on tomato roots. As shown in Figure 2, significant differences were observed among the CK (water), NB (medium), and G24 treatments in terms of the number, fresh weight, and dry weight of parasitic broomrape nodules per tomato plant.

As shown in Figure 2A–C, the CK group exhibited the highest level of parasitism, with a mean nodule number of 152.5 per plant, a fresh weight of 293.4 g per plant, and a dry weight of 33.5 g per plant. The NB medium treatment group (148.3 nodules per plant, fresh weight 354.5 g, dry weight 36.4 g) showed slight increases in fresh and dry weights and a slight decrease in nodule number compared with the CK group, but the differences were not statistically significant (*p* > 0.05), indicating that the NB medium itself exerted no observable inhibitory effect on broomrape parasitism.

In contrast, inoculation with strain G24 suppressed broomrape parasitism. The G24 treatment group showed the lowest values across all measured parameters, with a nodule number of only 46.6 per plant, a fresh weight of 32.2 g per plant, and a dry weight of 3.8 g per plant, representing reductions of 69.4%, 89.0%, and 88.6%, respectively, compared with the CK group. These reductions were statistically significant (*p* < 0.05) relative to both the CK and NB groups (Figure 2A–C).

Phenotypic observations (Figure 2D–F) were consistent with the quantitative data. The root surfaces of tomato plants in the CK and NB groups were covered with numerous *P. aegyptiaca* nodules. In contrast, the G24 treatment group exhibited significantly fewer parasitic broomrape nodules per tomato plant compared with the CK and NB groups, with one plant showing no broomrape parasitism at all. These results collectively demonstrated that strain G24 colonization effectively interfered with the parasitic infection process of *P. aegyptiaca* and significantly alleviated the damage caused by broomrape to tomato roots, highlighting its strong and stable biocontrol potential against root parasitic weeds.

#### 2.3.2. Effect of Endophytic Bacterium G24 on Tomato Growth and Physiological Traits

To evaluate the effect of *L. nimipressuralis* G24 colonization on tomato growth, we measured plant height, stem diameter, fresh weight, and total chlorophyll content under three treatments: non-inoculated control (CK), nutrient broth control (NB), and G24-inoculated (G24) plants.

**Figure 3:**
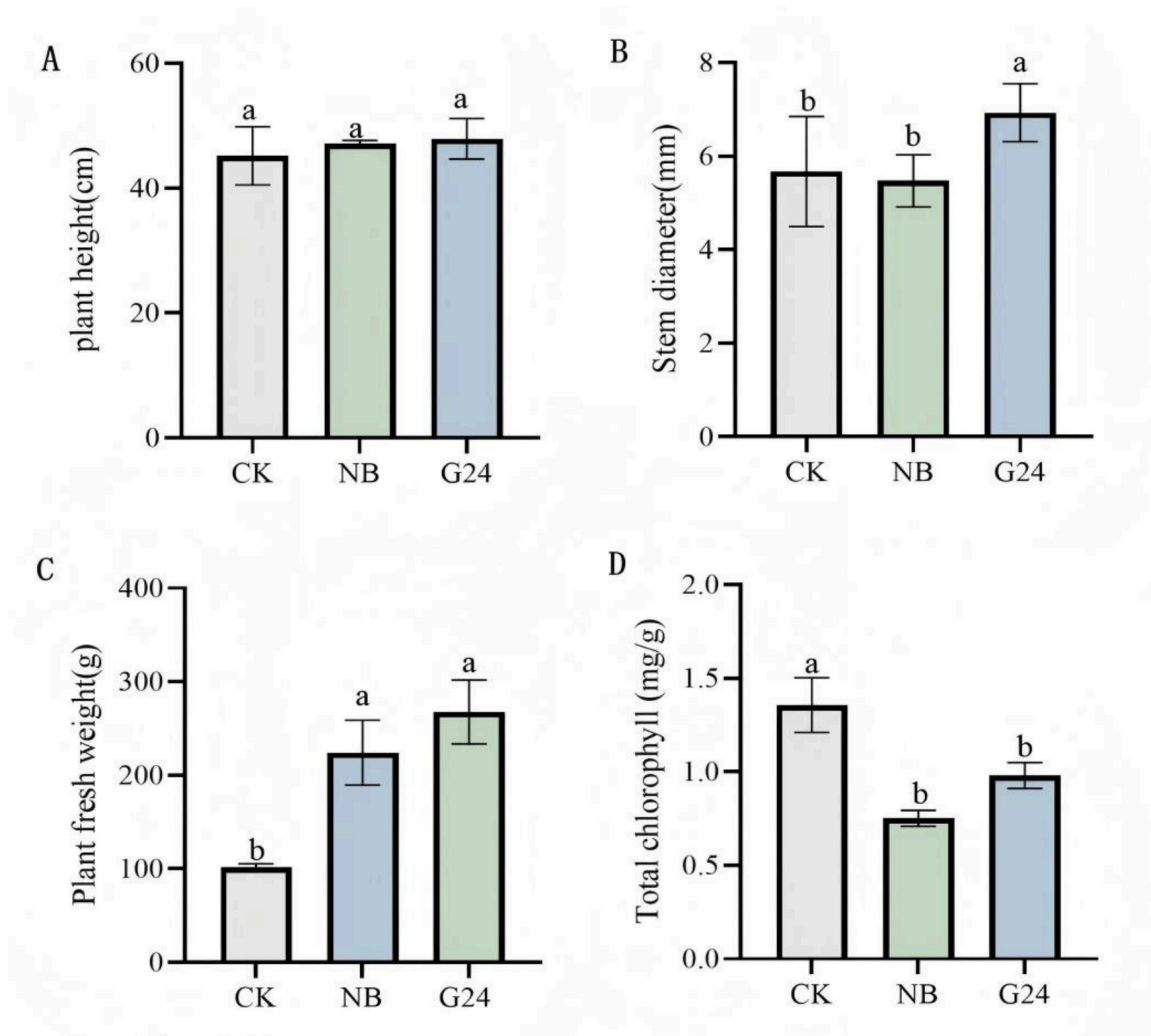
Effects of NB medium and strain G24 on tomato plant height (**A**), stem diameter (**B**), plant fresh weight (**C**) and total chlorophyll content (**D**). Different lowercase letters above bars indicate significant differences at *p* < 0.05 according to Duncan’s test. Error bars represent Standard Deviation (SD).

Plant height did not differ significantly among the three treatments at the measurement stage, with all plants reaching approximately 45–48 cm (*p* > 0.05; Figure 3A). In contrast, stem diameter was significantly greater in the G24 treatment (6.87 mm) compared to both CK (5.64 mm) and NB (5.46 mm), representing a 21.8% and 25.8% increase, respectively (*p* < 0.05; Figure 3B). No significant difference was observed between CK and NB for this trait. These results indicate that G24 inoculation exerts a pronounced growth-promoting effect on tomato plants, particularly in enhancing stem thickening.

The experimental results showed that the G24 treatment achieved a fresh weight of 265 g per plant, which was 2.65-fold higher than that of CK (100 g) and 1.18-fold higher than that of NB (225 g). The NB treatment also produced significantly greater fresh weight than CK (2.25-fold), indicating that the culture medium alone had a substantial growth-promoting effect on plants. However, no significant difference was observed in fresh weight between the G24 and NB treatments (*p* > 0.05; Figure 3C), suggesting that, under the conditions of this experiment, strain G24 did not confer any additional benefit in terms of fresh weight accumulation, and the increase in plant fresh weight was primarily attributable to the nutrients supplied by the medium.

Notably, total chlorophyll concentration per gram of leaf fresh tissue differed significantly across treatments (*p* < 0.05). The CK group exhibited the highest chlorophyll concentration (1.35 mg/g), which was significantly higher than the NB medium control (0.73 mg/g) and G24 inoculation treatment (0.96 mg/g). The chlorophyll concentration of the G24 group was numerically higher than that of the NB group, though this difference was not statistically significant. (*p* > 0.05; Figure 3D).

### 2.4. Identification of Strain G24

#### 2.4.1. Morphological Characteristics

Morphological observations showed that the single colonies of this strain were nearly round, milky white with smooth surfaces and irregular spreading margins. The cells were rod-shaped and Gram-negative.

#### 2.4.2. Physiological and Biochemical Characteristics

The physiological and biochemical properties of strain G24 are presented in Table 3. This strain was negative for Voges-Proskauer (V.P) test and hydrogen sulfide production, but positive for Methyl Red (MR) test and catalase activity. It was also identified as Gram-negative.

In terms of carbon source utilization, the strain could utilize a variety of carbon sources including sucrose, glucose and mannitol, whereas it failed to utilize creatine anhydrous. For amino acid utilization, histidine, proline and other common amino acids could not be used by this strain.

#### 2.4.3. Molecular Identification

**Figure 4:**
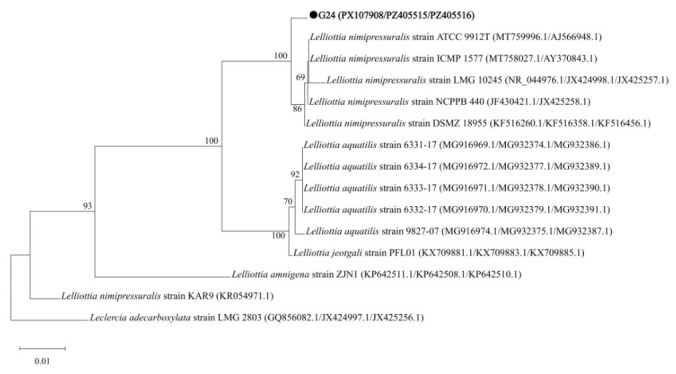
Phylogenetic analysis of *Lelliottia* and related genera based on combined *16S rDNA*, *gyrB*, and *rpoB* sequences using the neighbor-joining method with 1000 bootstrap replicates.

Based on the combined sequences of *16S rDNA*, *gyrB* and *rpoB* genes, a phylogenetic tree of strain G24 was constructed using the Neighbor-Joining (NJ) method in MEGA 11 software (Figure 4).

The results indicated that strain G24 clustered into a single clade with the type strains of *Lelliottia nimipressuralis* (DSMZ 18955 and LMG 10245), with bootstrap support values of 100% and 99% at the branching nodes. They shared the closest phylogenetic relationship with a minimal genetic distance. This clade formed a sister group to *Lelliottia jeotgali*.

Other species within the genus formed distinct monophyletic lineages. Five reference strains of *Lelliottia aquatilis* clustered together, with bootstrap support ranging from 67% to 100%, while *Lelliottia amnigena* formed an independent branch. *Leclercia adecarboxylata* strain LMG 2803 was selected as the outgroup, which clearly differentiated the phylogenetic positions of different species in the genus *Lelliottia*.

Combined with the multi-gene phylogenetic analysis, strain G24 was identified as *L. nimipressuralis*.

## 3. Discussion

Egyptian broomrape (*P. aegyptiaca*) is a destructive root parasitic weed that severely restricts the safe and sustainable production of tomato [24]. Conventional chemical control strategies are limited by obvious drawbacks, including environmental residue pollution, unstable field efficacy, and the progressive development of weed resistance [21,25,26]. Accordingly, microbe-based biocontrol has become a promising and eco-friendly strategy for the integrated management of parasitic weeds. At present, most available studies concerning broomrape biocontrol mainly focus on specialized pathogens isolated from diseased broomrape plants and indigenous rhizosphere soil microorganisms [16,21,26]. In contrast, research on the biocontrol potential of crop endophytic bacteria against *P. aegyptiaca* remains limited, and high-quality endophytic resources with both weed-suppressive and plant growth-promoting capacities are particularly scarce [27]. In this study, an endophytic bacterial strain *L. nimipressuralis* was isolated from healthy tomato tissues. Combined in vitro germination assays and greenhouse pot experiments systematically verified that this strain could significantly inhibit seed germination and parasitism of *P. aegyptiaca*, while effectively promoting increases in tomato stem diameter. This study confirms the dual potential of *L. nimipressuralis* in biocontrol and plant growth promotion, providing a novel endophytic microbial resource for the green prevention and control of broomrape.

Taxonomic identification confirmed this biocontrol strain as *L. nimipressuralis*, which has been deposited in the Guangdong Microbial Culture Collection Center (GDMCC) under the accession number 67314. Notably, the strain was preliminarily classified as *L. amnigena* relying merely on *16S rRNA* gene sequencing in the early stage, which was performed prior to the pot trials. Identification based on *16S rRNA* gene sequencing is reliable for genus-level classification, whereas multi-gene phylogenetic analysis enables accurate species-level identification [17]. In the present study, polygenic phylogenetic analysis was further performed after the pot trials to refine its taxonomic status, and the original identification was corrected to *L. nimipressuralis*.

*L. nimipressuralis*, belonging to the family Enterobacteriaceae, is an endophytic bacterium with strong environmental adaptability. Unlike pathogenic *L. nimipressuralis* strains that cause diseases in forest trees and other plants [28,29], the endophytic strain G24 isolated from healthy tomato plants in this study exhibited no evident pathogenic effects in pot experiments, and no disease lesions or symptomatic injuries were detected on any of the tested host plants, which demonstrates that this strain lacks virulence under the current experimental conditions. Nevertheless, we recognize that merely recording an absence of visible symptoms in pot trials cannot substitute a standardized pathogenicity assay. Further multi-condition trials covering broader host ranges and environmental gradients are therefore required to comprehensively evaluate the agricultural biosafety of strain G24. Previous studies have demonstrated that *Lelliottia* species generally possess multiple beneficial properties, including phosphorus solubilization, bioactive metabolite secretion, and the regulation of plant physiological metabolism [19,30,31]. These traits facilitate nutrient absorption and activate plant defense responses, which are consistent with our results that strain G24 significantly improved tomato stem diameter. In addition, strain *L. nimipressuralis* did not inhibit the vertical growth of tomato plants but effectively optimized plant growth performance. These findings indicate that this endophytic strain exerts growth-promoting effects without interfering with normal plant development. However, the pot experiment used only a single concentration (3.0 × 10^9^ CFU/mL), leaving the minimum effective concentration, optimal application rate, and potential phytotoxicity at higher doses unknown. Additionally, biosafety was assessed only on tomato; effects on other crops, non-target plants, and beneficial organisms remain untested. Future studies should include broader crop pathogenicity tests, ecotoxicological assessments, and multi-location field trials before field application can be recommended.

Beyond its growth-promoting effects, we further demonstrated that the fermentation supernatant of *L. nimipressuralis* exhibited strong inhibitory activity against broomrape seed germination, suggesting that this strain may suppress parasitic weeds through extracellular production or secretion of bioactive metabolites, which represents a typical functional mode of endophytic biocontrol bacteria [32]. Compared with conventional soil microorganisms, endophytic bacteria can stably colonize internal plant tissues and occupy exclusive ecological niches [33]. Such colonization enables endophytes to stably produce and release inhibitory substances without being severely affected by complex soil environments or competition from indigenous microbes [34]. Given that *L. nimipressuralis* significantly inhibited broomrape seed germination, it is reasonable to hypothesize that this strain may also interfere with subsequent haustorium formation and parasitism establishment. However, direct evidence for haustorium inhibition was not obtained in the present study, and this possibility warrants dedicated investigation in future work.

G24 was first applied at Day 7 post-transplantation, when roots were expected to be established and secreting stimulants. In vitro, seeds received stimulants from the start and germinated within 7–14 days. As the actual timing of stimulant release and germination was not monitored, some seeds may have germinated before the first application, possibly underestimating efficacy. Future applications should be prophylactic, before or at the germination window onset, with multiple shorter-interval applications. Time-course studies are needed to determine the optimal schedule.

NB medium alone showed differential effects on *P. aegyptiaca* seed germination across experimental systems: no inhibition in sterile in vitro assays, but approximately 24% reduction in the rhizosphere Petri dish assay containing tomato root tissues. This discrepancy likely arises from root-associated microorganisms. NB medium may have stimulated these microbes to produce inhibitory substances or modify the local microenvironment, indirectly suppressing germination. Such microbe-mediated effects would not occur in sterile, root-free assays. This suggests that the inhibitory contribution of NB medium itself should be interpreted with caution in plant-tissue assays, and highlights the role of root-associated microbiomes in modulating broomrape germination.

G24 inoculation boosted tomato vegetative growth (thicker stems, increased biomass driven by leaf expansion), accompanied by reduced leaf chlorophyll concentration (mg g^−1^ FW), consistent with a biomass dilution effect. As leaf biomass accumulates rapidly, chlorophyll pigments distributed across larger leaf areas inevitably lead to lower pigment concentration per gram of fresh leaf tissue [35,36]. This growth dilution phenomenon is widely reported in plants with accelerated vegetative growth, where expanded leaf mass dilutes photosynthetic pigment and nutrient concentrations without suppressing the total biosynthesis of chlorophyll at the whole-plant scale [37,38]. Notably, although a slight reduction in chlorophyll content was observed in tomato leaves following *L. nimipressuralis* inoculation, the overall biomass and growth vigor of the plants were significantly enhanced, indicating that the photosynthetic efficiency per unit leaf area remained sufficient to support robust plant growth. However, since we measured whole-plant fresh weight without separating leaf tissues, total chlorophyll per plant could not be quantified to verify this dilution effect. Collectively, *L. nimipressuralis* acts as a beneficial endophytic strain that effectively promotes tomato seedling growth without incurring adverse physiological effects on the host plants.

In conclusion, this study demonstrates that endophytic *L. nimipressuralis* exhibits biocontrol activity against *P. aegyptiaca* and promotes tomato growth, expanding the resource library for parasitic weed control and addressing the research gap on *Lelliottia* in broomrape management. However, several limitations remain: only phenotypic functions were verified; the active metabolites, colonization dynamics, and molecular mechanisms underlying tomato resistance are unclear; and colonization after rhizosphere irrigation was not confirmed. Future studies will focus on identifying key metabolites, elucidating action mechanisms, and conducting field trials to validate practical efficacy, ultimately supporting the development of *L. nimipressuralis* as a multifunctional microbial agent for green broomrape control in tomato cultivation.

## 4. Materials and Methods

### 4.1. Experimental Materials

We collected *P. aegyptiaca* seeds used in this study from processing tomato fields in Jimsar County, Changji Prefecture, Xinjiang, China. The collected seeds were then sieved, thoroughly mixed to ensure homogeneity, and stored in glass containers under cool, dry conditions until use. For each experiment, we randomly assigned seeds to treatments and experimental replicates.

The tomato cultivar used in this study was Xinhong 55, which was provided by the Institute of Fruits and Vegetables, Xinjiang Academy of Agricultural Sciences, Xinjiang, China.

We purchased strigolactone analog GR24 from Shanghai Macklin Biochemical Co., Ltd. (Shanghai, China).

### 4.2. Isolation of Endophytic Bacteria from Tomato

We collected roots, stems and leaves of healthy tomato plants in June 2024 from processing tomato fields in Jimsar County, Changji Prefecture, Xinjiang, China, with a total of 68 samples collected. The samples were cut into small pieces of approximately 0.5 cm in length, surface-sterilized with 75% ethanol for 1.5 min, treated with 1% sodium hypochlorite (available chlorine) for 8 min, and rinsed three times with sterile water [39]. To validate the surface sterilization efficacy, we spread the final rinse water onto nutrient agar (NA) plates and incubated them at 28 °C for 3 days; no microbial growth was observed, confirming effective elimination of epiphytic microorganisms and endophytic origin of the subsequent isolates. We then placed the sterilized tissues into a sterile mortar with sterile water, ground them thoroughly, and streaked the homogenate onto NA solid medium using a loop. We incubated the plates at 28 °C until single colonies formed, picked individual colonies with distinct morphological characteristics, and purified them by repeated streaking on NA medium. Two consecutive rounds of isolation were performed. Finally, we numbered the purified strains and preserved them for subsequent use.

### 4.3. Screening of Bacterial Strains with High Inhibitory Activity Against P. aegyptiaca Seed Germination

#### 4.3.1. Surface Sterilization of *P. aegyptiaca* Seeds

Seeds of *P. aegyptiaca* were first surface-sterilized with 75% ethanol for 1.5 min, followed by 1% sodium hypochlorite (available chlorine) for 12 min. The seeds were rinsed three times with sterile water and air-dried in a laminar flow cabinet for later use.

#### 4.3.2. Preparation of Bacterial Suspensions

Strains were preserved as glycerol stocks at –80 °C and maintained on NA slants at 4 °C. Before each experiment, strains were activated on NA plates at 28 °C for 24–48 h, and a single colony was cultured in NB at 28 °C and 180 r/min for 24 h to prepare the seed culture. Bacterial suspensions were adjusted to the required concentrations via serial dilution and plate counting, and all strains were standardized to the same CFU/mL before use.

#### 4.3.3. Primary Screening

The bacterial suspension was serially diluted 100-fold with sterile water. Two layers of sterile filter paper were placed in each well of a 24-well plate. Approximately 60–70 *P. aegyptiaca* seeds were placed in each well. A total of 150 μL GR24 solution (0.05 mg/mL) was added first, followed by 150 μL diluted bacterial suspension [40]. For the control group, an equal volume of the mixture of sterile water and GR24 solution was applied. Each treatment consisted of four replicates. All plates were incubated at 26 °C in darkness for 7 days, and seed germination was observed under a stereomicroscope. The germination rate of *P. aegyptiaca* seeds was calculated using the formula below.

Germination rate (%) = (germinated seeds/total seeds) × 100%

#### 4.3.4. Secondary Screening

The bacterial suspension was serially diluted to 200-, 500- and 1000-fold with sterile water. The experimental procedures were consistent with those described for primary screening.

### 4.4. Assay on the Inhibitory Effect of Strain G24 Fermentation Broth on Seed Germination of P. aegyptiaca in Tomato Rhizosphere

Four treatments were designed in this experiment: undiluted fermentation broth of strain G24, fermentation supernatant of strain G24, Nutrient Broth (NB) as negative control, and distilled water as blank control (CK), each with six replicates. The fermentation broth of strain G24 was prepared following the same procedure as described in Section 4.3.2, with a final viable cell concentration of 2.72 × 10^8^ CFU/mL. The cell-free supernatant was obtained by centrifugation (10,000× *g*, 15 min, 4 °C) and 0.22 µm filtration; plating on NA and incubation at 28 °C for 48 h confirmed the absence of bacterial cells. Tomato seedlings with 4–5 true leaves were gently removed from plug trays, thoroughly rinsed of root soil, and placed in Petri dishes lined with absorbent cotton and two layers of filter paper, to which 3 mL of the respective solutions (undiluted G24 fermentation broth at 2.72 × 10^8^ CFU/mL, G24 fermentation supernatant, NB medium, or distilled water) were added. Subsequently, 15 mg of *P. aegyptiaca* seeds were evenly sown on the filter paper of each dish, and all Petri dishes were wrapped with aluminum foil and incubated in a light incubator. After 15 days of incubation, a 1.5 cm × 1.5 cm area was marked within the *P. aegyptiaca* seed distribution zone of each dish; for each treatment, seed germination was assessed in five independent replicates, with the total sampled area across replicates covering approximately 30% of the total seed distribution area. The seed germination rate of rhizospheric *P. aegyptiaca* was calculated within the designated area according to the formula described in Section 4.3.2.

### 4.5. Pot Assay

The pot assay was conducted in the greenhouse at Anningqu Comprehensive Experimental Station, Xinjiang Academy of Agricultural Sciences, Urumqi, Xinjiang, China.

#### 4.5.1. Preparation of Strain Fermentation Broth

The preserved test strain was streaked onto NA medium and incubated at 28 °C for 24 h. A single colony was picked and inoculated into a test tube containing 5 mL of NB, then incubated with shaking at 28 °C and 180 r/min for 24 h to obtain the primary seed culture. Subsequently, the primary seed culture was transferred into a 3000 mL Erlenmeyer flask filled with 1500 mL of NB and incubated at 28 °C and 180 r/min for 48 h to obtain the secondary fermentation broth, whose viable cell concentration was determined to be 3.0 × 10^9^ CFU/mL via the plate counting method. The secondary fermentation broth was kept in the original flask for subsequent use.

#### 4.5.2. Application of Strain Fermentation Broth

Three treatments were set up in this experiment: G24 fermentation broth treatment, NB medium control, and distilled water control (CK), each with six replicates. The potting soil was prepared by mixing field soil free of *P. aegyptiaca* contamination and peat at a ratio of 10:1, and each pot (26 cm in diameter) was filled with soil to approximately one-third of its volume; then 100 mg of *P. aegyptiaca* seeds were fully mixed with 50 g soil and evenly spread on the soil surface. Tomato seedlings with 5–6 true leaves were transplanted into the pots, one seedling per pot, and all seedlings were thoroughly watered for root establishment and acclimated under suitable conditions. Root irrigation treatments started 7 days after transplanting, with each pot receiving 100 mL of G24 fermentation broth for the bacterial treatment, 100 mL of NB medium for the medium control, or 100 mL of distilled water for the blank control. Irrigation was performed once every 7 days for three consecutive times, and regular watering and management were maintained throughout the experimental period.

#### 4.5.3. Investigation of Pot Experiment

Tomato plant height and stem diameter were measured at 30 days after transplanting. Meanwhile, 1–2 fully expanded functional top leaves without disease spots, insect damage and mechanical injury were collected from each treatment. The leaf samples were wrapped with tin foil, labeled, frozen rapidly in liquid nitrogen, and stored at −80 °C in a refrigerator for subsequent chlorophyll determination. The total chlorophyll content was detected using the ethanol extraction-spectrophotometric method with a commercial assay kit G0601W, Suzhou Grace Biotechnology (Suzhou, China). Briefly, the extraction buffer was prepared by mixing 95% ethanol and distilled water at a volume ratio of 95:5. A total of 0.1 g of fresh leaf tissue was cut into pieces, rinsed with distilled water, fully ground with 1 mL extraction buffer and approximately 50 mg of reagent I under dark or low-light conditions, and then transferred into a 10 mL glass test tube. The mortar was thoroughly rinsed with extraction buffer, and all eluents were combined and diluted to a final volume of 10 mL. The samples were incubated in darkness for 3 h until the tissue residues were completely whitened. Subsequently, 200 μL of the extracting solution and 200 μL of blank extraction buffer were added to a 96-well plate as the measurement group and blank group, respectively. The absorbance values at 665 nm and 649 nm were determined. Samples with absorbance values exceeding 1.0 were appropriately diluted before detection, and the dilution factor was adopted for final calculation. The contents of chlorophyll a, chlorophyll b and total chlorophyll were calculated according to the corresponding standard formulas. At 65 days after transplanting, all tomato plants were harvested, and the root surface soil was carefully cleaned. The plant fresh weight, as well as the number, fresh weight and dry weight of *P. aegyptiaca* nodules were determined for each treatment.

### 4.6. Identification of Strain G24

Morphological characteristics were observed via Gram staining and spore staining. Physiological and biochemical characteristics were identified according to the Manual for Systematic Identification of Common Bacteria and Archaea [41].

Genomic DNA of strain G24 was extracted using SK8255 Ezup Column Bacterial Genomic DNA Extraction Kit (Sangon Biotech (Shanghai) Co., Ltd.). Three gene fragments, namely *16S rDNA* (27-F: 5′-AGAGTTTGATCCTGGCTCAG-3′; 1492-R: 5′-CGGTTACCTTGTTACGACTTC-3′), *gyrB* (UP1: 5′-GAAGTCATCATGACCGTTCTGCAYGCNGGNGGNAARTTYGA-3′; UP2r: 5′-AGCAGGGTACGGATGTGCGAGCCRTCNACRTCNGCRTCNGTCAT-3′) and *rpoB* (*rpoB*-F: 5′-GCAAGGTTAAGCGATTTACCG-3′; *rpoB*-R: 5′-CCTGTTGATTTGTTGATTTG-3′), were amplified by PCR.

The PCR reaction system totaled 50 μL, containing 25 μL of 2× PCR Mix, 1 μL of forward primer (10 μmol/L), 1 μL of reverse primer (10 μmol/L), 1 μL of DNA template, and sterile ddH_2_O added to bring the final volume to 50 μL. The thermal profile was set as follows: initial denaturation at 94 °C for 5 min; 35 cycles of denaturation at 94 °C for 30 s, annealing at 54 °C for 30 s, and extension at 72 °C for 90 s; followed by a final extension at 72 °C for 10 min.

PCR products were detected by 2.0% agarose gel electrophoresis. Specific target bands were excised for gel extraction and purification using the SanPrep Column DNA Gel Extraction Kit (Sangon Biotech, Shanghai, China; Cat. No. B518131-0050). For every 0.1 g of gel, 300 μL of Binding Buffer (Solution A) was added, and the mixture was incubated in a water bath at 60 °C until the gel was completely dissolved. After adding and mixing with 50 μL of Washing Buffer (Solution B), the solution was transferred to an adsorption column and kept at room temperature for 5 min. The column was centrifuged at 12,000 r/min for 1 min, and the waste liquid was discarded. Subsequently, 500 μL of 80% ethanol was added for washing, followed by centrifugation at 12,000 r/min for 1 min, and the washing step was repeated once. The column was then spun empty at 12,000 r/min for 5 min to remove residual ethanol and air-dried at room temperature for 5–8 min. Finally, 30 μL of sterile ddH_2_O was added to the column; after standing for 10 min, the sample was centrifuged at 13,000 r/min for 3 min to elute purified DNA. A 3 μL aliquot of the purified product was verified by agarose gel electrophoresis.

The purified DNA samples were sent to Sangon Biotech (Shanghai) Co., Ltd. for sequencing. The obtained sequences were subjected to NCBI online BLAST (BLAST+ version 2. 17. 0+) homology search against the NCBI database. BLASTn searches against the NCBI nr database identified sequences for phylogenetic analysis, selected from top hits (>98% similarity), related Enterobacteriaceae genera, type strains, and available pathogenic *L. nimipressularis* isolates. The concatenated sequences of *16S rDNA*, *gyrB* and *rpoB* were used to construct a phylogenetic tree via MEGA 11 software.

### 4.7. Statistical Analysis

All experiments were conducted on at least three independent occasions with separately prepared cultures and freshly prepared reagents, ensuring true biological replication. Each independent experiment included three technical replicates per treatment. Values are presented as mean ± standard deviation (SD) from at least three biological replicates.

All data were tested for normality and homogeneity of variances using Shapiro–Wilk and Levene’s tests, respectively. As the assumptions for parametric ANOVA were violated for most datasets, non-parametric Kruskal–Wallis tests were performed, followed by Dunn’s post hoc test with Bonferroni correction for multiple comparisons. For datasets that met the assumptions of normality and variance homogeneity, one-way ANOVA followed by Duncan’s multiple range test was applied. All statistical analyses were conducted using SPSS version 25.0, and statistical significance was set at *p* < 0.05. Different lowercase letters within the same column indicate significant differences among treatments.

## 5. Conclusions

In this study, we isolated and identified an endophytic bacterial strain G24 from healthy tomato tissues as *L*. *nimipressuralis*, and systematically evaluated its biocontrol potential against the root parasitic weed *P*. *aegyptiaca*. Both in vitro germination assays and greenhouse pot experiments confirmed that strain G24 effectively inhibits seed germination and suppresses parasitic nodule formation on tomato roots, by up to 69.4% for nodule number and over 88% for biomass (fresh weight: 89.0%; dry weight: 88.6%). These results suggest that the active compounds responsible for inhibition are likely extracellular metabolites produced or released by the strain. In addition to its biocontrol function, G24 inoculation significantly promotes tomato vegetative growth, as reflected by increased stem diameter, without causing any detectable phytotoxicity or growth retardation. As a native endophytic bacterium of tomato, *L. nimipressuralis* G24 exhibits strong host compatibility and dual functionality, acting both as a suppressor of parasitic weeds and a promoter of plant growth. These findings enrich the microbial resource library for parasitic weed biocontrol and address the research gap regarding the application of the genus *Lelliottia* in broomrape management. Future work will focus on identifying the key active metabolites, elucidating the molecular mechanisms underlying its biocontrol and growth-promoting effects, and validating its field efficacy under practical agricultural conditions. Overall, *L. nimipressuralis* G24 represents a promising and sustainable biocontrol agent for integrated management of *P. aegyptiaca* in tomato production systems.

## Figures and Tables

**Table 1:** Inhibitory effects of 100-fold diluted fermentation broth from certain bacterial strains on *P. aegyptiaca* seed germination.

Strain No.	Germination Rate (%)	Relative Inhibition Rate (%)
GT-1	0.00 ± 0.00 †	100.00 ± 0.00 †
GT-2	0.00 ± 0.00 †	100.00 ± 0.00 †
GT-3	0.00 ± 0.00 †	100.00 ± 0.00 †
JTB-4	0.00 ± 0.00 †	100.00 ± 0.00 †
G24	0.00 ± 0.00 †	100.00 ± 0.00 †
TG4-2	93.78 ± 2.64 ab	6.19 ± 2.71 cd
TG5-1	95.84 ± 0.50 ab	4.13 ± 0.51 e
TJ2-1	90.07 ± 9.24 abc	9.90 ± 9.48 b
TY2-1	91.99 ± 2.79 abc	7.98 ± 2.86 c
TG4-3	95.78 ± 1.74 ab	4.19 ± 1.79 e
TG6-2	88.09 ± 4.92 bc	11.88 ± 5.05 b
TJ4-1	93.21 ± 3.26 ab	6.76 ± 3.34 cd
TY5-1	96.31 ± 1.94 a	3.66 ± 1.99 e
TG2-2	89.90 ± 4.65 abc	10.07 ± 4.77 b
TG6-3	85.59 ± 5.51 c	14.38 ± 5.65 b
TY5-2	95.81 ± 0.78 ab	4.16 ± 0.80 e
TY1-1	93.32 ± 2.43 ab	6.65 ± 2.49 cd
NB	91.82 ± 3.65 abc	
CK	97.46 ± 1.29 a	

† Treatments with 0.00 ± 0.00% germination and 100.00 ± 0.00% inhibition were excluded from analysis due to zero variance and are described as complete inhibition. For other treatments, different lowercase letters indicate significant differences (*p* < 0.05, Kruskal–Wallis with Dunn’s test). NB: nutrient broth; CK: blank control (CK inhibition defined as 0%, excluded from analysis). Values are mean ± SD (*n* = 4).

**Table 2:** Inhibitory effects of fermentation broths from different strains on seed germination of *P. aegyptiaca* at different dilution multiples.

Strain No.	Dilution Multiple	Germination Rate (%)	Relative Inhibition Rate (%)
GT-1	200	92.75 ± 3.29 ab	6.28 ± 3.39 c
	500	93.75 ± 3.29 ab	5.26 ± 3.39 c
	1000	96.06 ± 2.78 a	2.93 ± 2.89 c
GT-2	200	86.37 ± 4.11 bc	12.72 ± 4.20 b
	500	92.87 ± 3.19 ab	6.15 ± 3.29 c
	1000	96.34 ± 1.76 a	2.65 ± 1.90 c
GT-3	200	92.87 ± 4.30 ab	6.15 ± 4.39 c
	500	97.67 ± 1.56 a	1.30 ± 1.72 c
	1000	97.95 ± 1.91 a	1.02 ± 2.05 c
JTB-4	200	83.91 ± 3.37 c	15.21 ± 3.46 a
	500	86.32 ± 3.94 bc	12.77 ± 4.03 b
	1000	95.44 ± 2.63 a	3.56 ± 2.74 c
G24	200	0.00 ± 0.00 †	100.00 ± 0.00 †
	500	0.00 ± 0.00 †	100.00 ± 0.00 †
	1000	0.00 ± 0.00 †	100.00 ± 0.00 †
NB		96.58 ± 0.60 a	
CK		98.96 ± 0.69 a	

† Treatments with 0.00 ± 0.00% germination and 100.00 ± 0.00% inhibition (G24 at all dilutions) were excluded from statistical analysis due to zero variance and are described as complete inhibition. For the remaining treatments, different lowercase letters within a column indicate significant differences (*p* < 0.05, Kruskal–Wallis test followed by Dunn’s test). NB: nutrient broth control; CK: blank control (distilled water); CK inhibition was defined as 0% and excluded from analysis. Values are mean ± SD (*n* = 4).

**Table 3:** Partial physiological and biochemical characteristics of strain G24.

Test Index	Result	Test Index	Result
Voges-Proskauer (V.P)	−	Sucrose	+
Methyl Red (MR)	+	Glucose	+
Catalase	+	Creatine anhydrous	−
Gram staining	−	Histidine	−
Hydrogen sulfide (H2S) production	−	Proline	−
Mannitol	+	Isoleucine	−
Glycerol	+	Methionine	−
D-Cellobiose	+	Valine	−
Maltose	+	Acetic acid	+

## Data Availability

All the data related to this project are presented here.
